# Growth inhibitory effect of *Leptospermum scoparium* (manuka) chloroform extract on breast and liver cancer cell lines 

**DOI:** 10.5455/javar.2024.k769

**Published:** 2024-06-04

**Authors:** Mohammed Al-Zharani

**Affiliations:** Biology Department, College of Science, Imam Mohammad Ibn Saud Islamic University (IMSIU), Riyadh, Saudi Arabia

**Keywords:** Antiproliferative, apoptosis, *Leptospermum scoparium*, MTT, LDH, MCF-7, GC-MS

## Abstract

**Objective:**

Research has demonstrated that *Leptospermum scoparium* possesses various therapeutic benefits. This study set out to determine whether or not *L. scoparium* extracts had any effect on the ability of HepG2 and MCF-7 breast cancer cells to survive.

**Materials and Methods:**

The antiproliferative activity of *L. scoparium* extracts was explored using 3-(4,5-dimethylthiazol-2-yl)-2,5-diphenyltetrazolium bromide and lactate dehydrogenase assays. The most active fraction was selected to investigate its effects on apoptosis induction using flow cytometry and quantitative real-time polymerase chain reaction. The constituents of this fraction were characterized using GC-MS analysis.

**Results:**

Research demonstrated that the chloroform fraction of *L. scoparium* (LSCF) significantly impacted the HepG2 and MCF-7 cancer cell lines. Treatment with LSCF led to a notable rise in both early and late apoptotic cells. Furthermore, there was an upregulation in the mRNA levels of P53, Bax, and caspases, while the expression of Bcl-2 mRNA saw a decrease. The analysis of LSCF revealed the primary components to be cis-calamenene, beta-eudesmol, cyclododecane, and alpha-muurolene.

**Conclusion:**

The study showed the promising antiproliferative activity of *L. scoparium*, suggesting its potential application for cancer treatment.

## Introduction

Cancer is a major concern in public health since it is the main killer on a global scale. The initiation of cancer is marked by changes to the genetic material in normal cells, which interfere with the cellular signaling pathways that control cell growth and programmed cell death [1,2]. Conventional cancer treatments often have adverse side effects, making alternative therapies necessary [3]. Natural products, such as chemotherapeutic agents derived from plants, are gaining popularity due to their relative safety.

**Figure 1.**
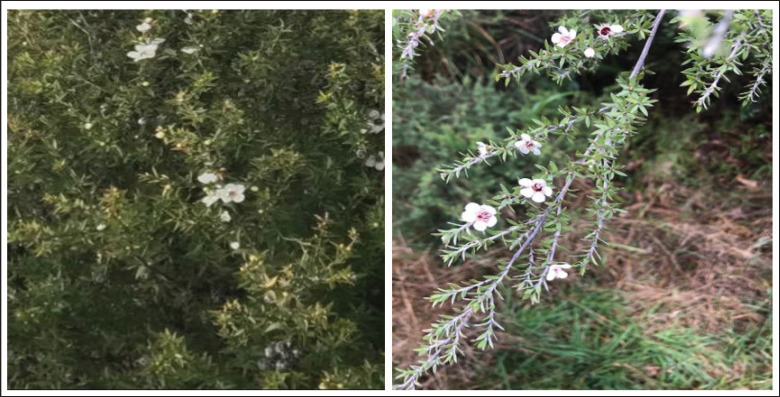
Manuka tree, *L. scoparium* (Accessed on July 2019).

*Leptospermum scoparium*, commonly referred to as manuka or tea tree, is a tree of varying heights originating from New Zealand and is part of the *Myrtaceae* family (Fig. 1). This family is found across various regions, such as Australia and Southeast Asia, encompassing around 133 genera and over 3,800 species. Manuka has a long history of traditional use, including the boiling of its leaves for tea and using manuka oil as an antiseptic [4]. The essential oil produced by *L. scoparium* has antimicrobial and antioxidant properties [5]. However, manuka honey (MH), made from the nectar of its flowers, is the most prized product of the species, as it has numerous therapeutic properties, including wound healing and treatment of fungal/bacterial infections, skin ulcers, and gastrointestinal diseases [6].

Numerous laboratory and animal studies indicate that MH can suppress the growth of different cancer cell types [7]. This anti-cancer activity is attributed to its ability to promote apoptosis and cause DNA fragmentation [8]. Its therapeutic properties are due to the components of plant origin, such as flavonoids and polyphenols. Although MH is widely used for medical purposes [9], its most significant application is its anticancer activity [10,11]. The use of manuka leaf extract in treating breast and liver cancer cell lines shows promise [12], but more research is needed to establish its safety and efficacy.

Published research on the cytotoxic effects of *L. scoparium* species is scarce. Consequently, this research seeks to explore the cytotoxic and apoptotic effects of extracts from the manuka plant on MCF-7 and HepG2 cancer cell lines, as well as to determine the specific phytochemicals that may contribute to these effects.

## Materials and Methods

### Collecting plants and processing their extracts

*Leptospermum scoparium* samples were collected between June and July 2019 from the South Island of New Zealand. The identification of the *L. scoparium* specimen was confirmed by a taxonomist from King Saud University’s. Next, the plant matter was finely powdered. It was then extracted using the Soxhlet device from 50 gm of this powdered substance. Hexane, chloroform, ethyl acetate, and methanol were among the solvents used for this task. The extract was concentrated by running it through a rotary evaporator set to 45°C.

### Cell viability test using 3-(4,5-dimethylthiazol-2-yl)-2,5-­diphenyltetrazolium bromide (MTT) 

Cell survival following the methodology previously detailed [13], the antiproliferative properties of *L. scoparium* extracts were assessed using the MTT reduction test. Afterward, a microplate reader calibrated to 540 nm was used to quantify the optical density.

**Figure 2.**
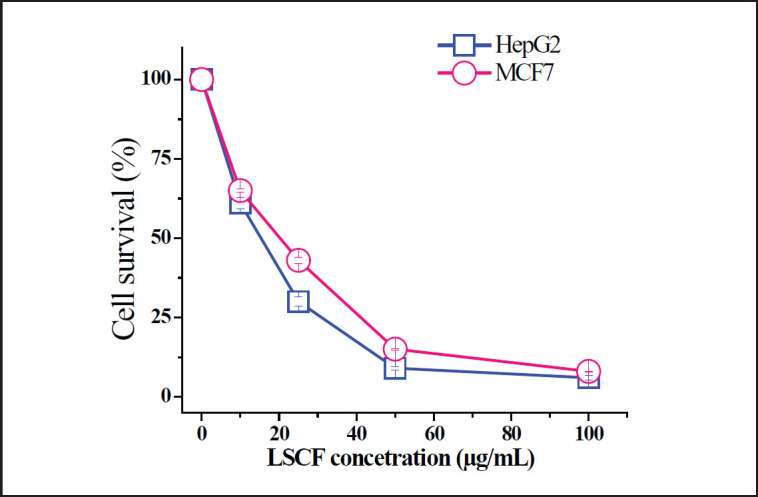
Effect of LSCF on HepG2 and MCF-7 cells using MTT assay. Cells were treated with different concentrations of extract for 48 h and the absorbance of the MTT formazan was determined at 540 nm in an ELISA reader. Data represent means ± SD.

### Release assay of lactate dehydrogenase (LDH)

LDH kits are being used to assess the release of LDH from cells. The levels of LDH in the treated samples were compared to those in the control samples, following the procedure outlined in the kit’s instructions.

**Figure 3.**
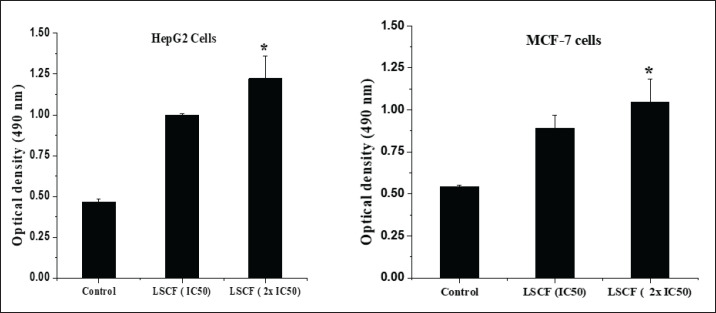
Cytotoxicity of LCSF on HepG2 and MCF-7 cells evaluated by LDH assay. LDH activity was determined at 490 nm in an ELISA reader. Values represent means ± SD (*p < 0.05) was considered significant compared to control) of three independent experiments carried out in triplicates.

### Apoptosis detection

After being treated for 24 h with a kit from BioLegend, USA, HepG2, and MCF-7 cell lines were subjected to an apoptotic staining experiment utilizing FITC Annexin V and PI. The flow cytometry system was used for the analysis, and the results were interpreted by CXP software.

### Quantitative real-time PCR (qRT-PCR)

Following exposure to IC_50_ and 2x IC_50_ of *L. scoparium* chloroform fraction (LSCF), RNA was extracted from both control and treated cells using Trizol TM reagent [14]. Afterward, cDNA was produced using a specialized kit (Invitrogen™, USA) called the SuperScript™ VILO™ Synthesis Kit. After that, we followed the protocol from the previous work [15] and ran qRT-PCR using a reaction mixture that included cDNA, SYBR Green, and particular primers. ΔCT = (CT gene of interest – CT internal control) was the formula used to determine the mRNA expression level, with glyceraldehyde-3-phosphate dehydrogenase serving as the internal reference.

### Mass spectrometry analysis (GC/MS)

GC/MS analysis of the *L. scoparium* chloroform extract. Spectra were compared between the extracts and libraries maintained by the NIST and WILEY Spectral to determine the components.

### Data analysis

The information is shown as the average ± standard deviation, based on a minimum of three measurements. The comparison of datasets was conducted using the *t*-test.

## Results

### MTT proliferation assay

The cytotoxic effect of various solvent fractions of *L. scoparium *was assayed on HepG2 and MCF-7 cells. The various concentrations were checked using an MTT assay and all fractions showed variable effects (Table 1). In comparison to all tested fractions, LSCF was the most active fraction with more adverse effects on HepG2 cells (IC_50_ = 15 µg/ml) followed by MCF-7 (IC_50_ = 20 µg/ml). Since LSCF exerted a remarkable anti-proliferation effect against both cells (Fig. 2), it was selected for further assays.

### LDH assay

LDH assay was employed to confirm the cytotoxic effect of LSCF. The results disclosed that LSCF treatments caused a higher release of LDH than non-treated cells. Figure 3 shows that the LCSF’s cytotoxicity was concentration-dependent and that both cell lines were significantly LDH-released at the IC_50_ concentration of the extract (*p* < 0.05).

### LCSF induces apoptosis in HepG2 and MCF-7 cells

**Figure 4.**
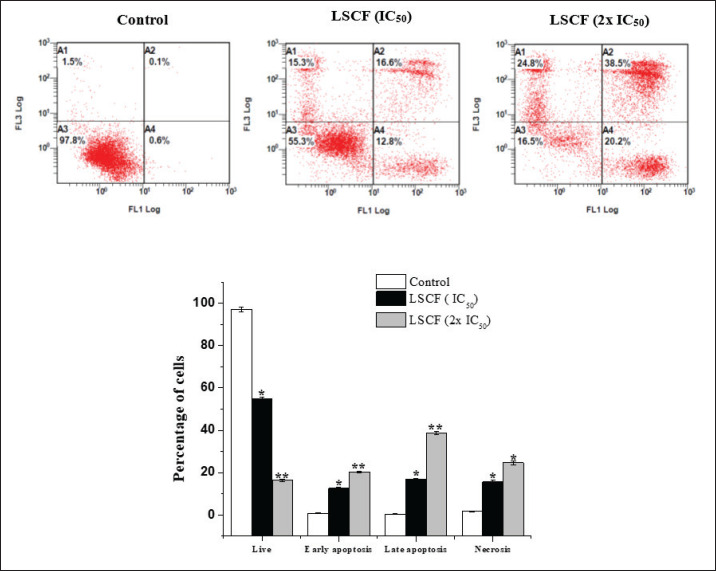
Effect of LSCF on HepG2 cells using annexin V-FITC/PI double staining. HepG2 cells were treated with 15 and 30 µg/ml for 24 h. (A) Hepg2 cells in different stages (A4) Early-stage apoptotic cells, (A3) viable cells (A2) cells in the late stage of apoptosis (A1) cells undergoing necrosis. (B) Apoptotic rate (%) of cell populations in different stages. Results are presented as the mean ± SD.

**Figure 5.**
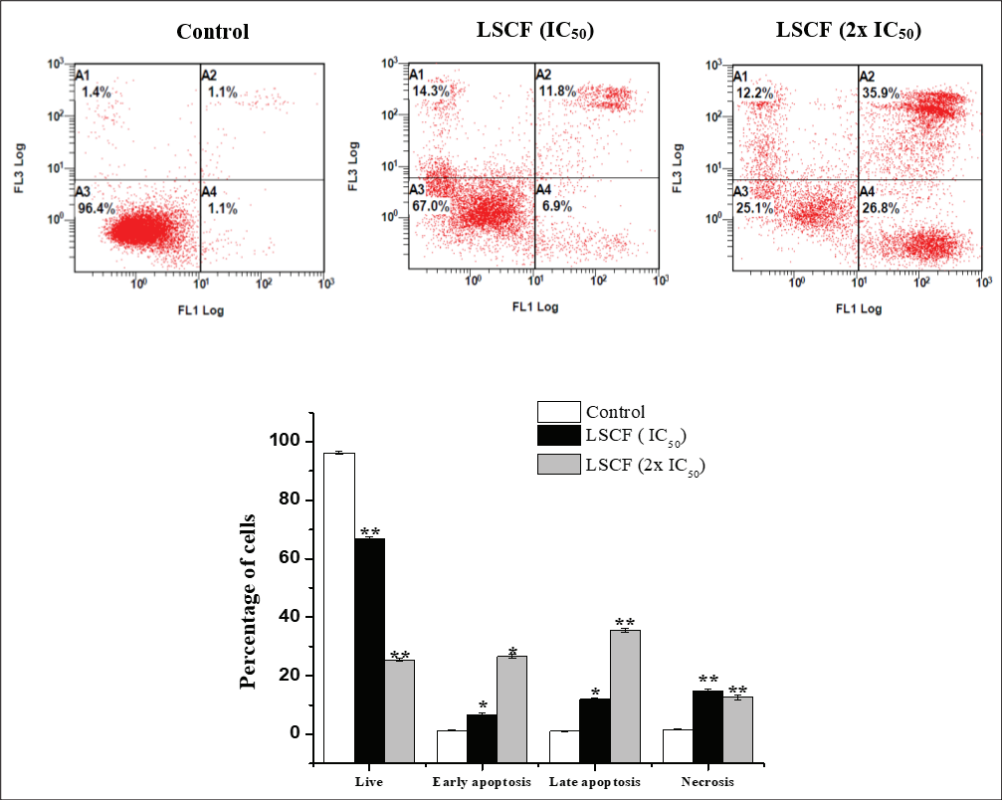
Effect of LSCF on MCF-7 cells using annexin V-FITC/PI double staining. MCF-7 cells were treated with 20 and 40 µg/ml) for 24 h. (A) (A4) Early-stage apoptotic cells, (A3) viable cells (A2) cells in late stage of apoptosis (A1) cells undergoing necrosis. (B) Apoptotic rate (%) of cell populations in different stages. Results are presented as the mean ± SD.

The association between the decrease in cell viability and the induction of apoptosis was examined in HepG2 and MCF-7 cells using FITC-Annexin V and PI labeling, respectively. According to our findings, LCSF caused cell death with only a small percentage of dead cells (Figs. 4 and 5). As shown in (Fig. 4), HepG2 cells treated with LCSF at15 and 30 µg/ml (IC_50_ and 2x IC_50_) displayed a dose-dependent increase of early (12.8% ± 0.4% and 20.2% ± 0.5%) and late apoptosis (16.6% ± 0.6% and 38.5% ± 0.9%) within 24 h of treatment, relative to the control. The same results were shown in MCF-7 (Fig. 5), when compared to control cells, early and late apoptotic cells increased dramatically to 6.9% ± 0.5% and 26.8% ± 0.7%, and 11.8% ± 0.4% and 35.9% ± 0.6%, respectively.

**Table 1.** IC_50_ values of *L. scoparium* different fractions against HepG2 and MCF-7 cancer cells.

Fraction	IC_50_ (µg/ml)	
	HepG2	MCF-7
Hexane	59.5 ± 1.5	49.1 ± 1.1
Chloroform	15 ± 0.3	20 ± 0.5
Ethyl acetate	188.8 ± 3.6	191.6 ± 2.2
Methanol	173 ± 3.3	162.5 ± 2.5
Doxorubicin	1.5 ± 0.2	1.4 ± 0.1

### Effect of LSCF on apoptosis-related gene expression levels

**Figure 6.**
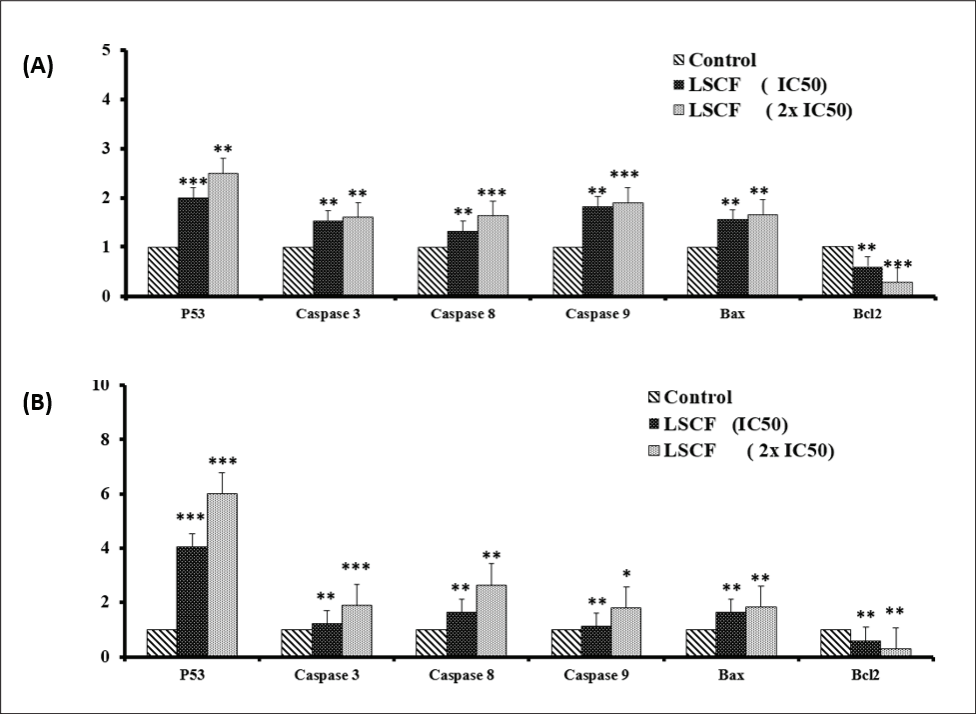
Effect of LSCF on the expression of apoptosis-associated genes in HepG2 and MCF-7 cells. (A) Relative expression of p53, and caspases 3, 8, 9, Bax, and Bcl2 genes in HepG2 cancer cells treated with 15 and 30 µg/ml concentrations for 24 h compared with the control (untreated cells). (B) Relative expression of the same genes in MCF-7 cancer cells treated with 20 and 40 µg/ml concentrations for 48 h. *p < 0.05, **p < 0.01, and ***p < 0.001 compared with the control (untreated cells). Data are presented as the mean ± standard deviation.

Levels of gene expression associated with apoptotic induction were also evaluated in HepG2 and MCF-7 cells treated with LSCF. Quantitative real-time polymerase chain reaction (RT-PCR) showed that levels of pro-apoptotic genes P53 and Bax rose dose-dependently while levels of anti-apoptotic gene Bcl-2 fell dramatically when treated cells were compared to untreated controls (Fig. 6). In addition, LCFS treatments resulted in up-regulation of caspases3, 8 and 9 mRNA expression levels compared to untreated cells (Fig. 6). Overall, our data indicated that LSCF inhibits HepG2 and MCF-7 cells proliferation through apoptosis pathway.

### LSCF GC-MS analysis

**Figure 7.**
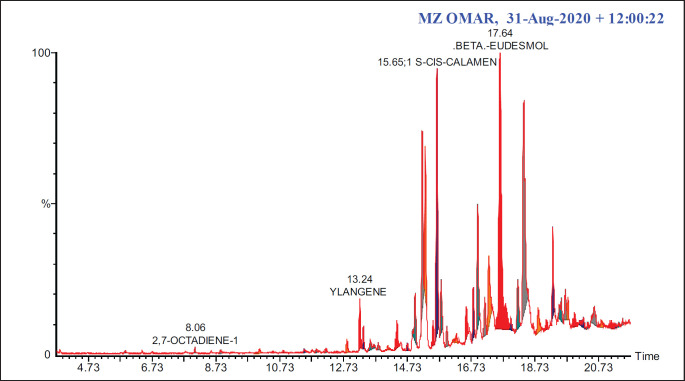
GC-MS spectra of LSCF.

In the LSCF, 25 primary components were identified using GC-MS data. Table 2 displays the retention time (RT), chemical formula, and molecular weight of these compounds. The peaks that showed the presence of some compounds are displayed in (Fig. 7). The major bioactive constituents identified were cis-calamenene (36.44%), beta-eudesmol (18.21%), cyclododecane (5.560%) and alpha-muurolene (3.62%) (Table 2).

## Discussion

There is increasing curiosity about the ­possibility of using natural products generated from plants to create new therapeutic cancer treatments, which is a major obstacle in modern medicine [16]. Examining the potential cytotoxic effects of *L. scoparium* fractions on HepG2 and MCF-7 cancer cell lines was the primary objective of this investigation. The ability of these fractions to induce apoptosis was verified using flow cytometry and quantitative real-time PCR. All tested fractions demonstrated an anti-proliferative effect that increased with concentration in both breast and liver cancer cells [17]. Strong inhibitory activity is indicated by an IC_50_ value of 30 µg/ml or lower, as per the criteria established by the ANC Institute [18,19]^19^]. The first experiments demonstrated that fractions of *L. scoparium* had strong inhibitory effects, with IC_50_ values of 15 µg/ml for HepG2 cells and 20 µg/ml for MCF-7 cells. Additionally, the LDH assay was used to evaluate the extent of cell membrane damage. This assay quantifies the release of this enzyme from the cytosol into the extracellular space upon cell death. The findings from both the LDH and MTT assays suggest that *L. scoparium* fractions possess notable cytotoxic properties [20,21]^21^]. Comparable results were also observed in cell lines of the lung cancerous cell treated with fractions from *Leptospermum flavescens*, a related species, in a similar experimental setup [22].

According to the study referenced by Afrin et al. [14], the analysis indicates that Strawberry tree honey (STH) possesses elevated concentrations of phenolics, flavonoids, amino acids, and proteins, along with stronger antioxidant activity, when compared to MH. Furthermore, both types of honey exhibit cytotoxic effects on colon cancer cells, which vary depending on the dose and duration of treatment. Nevertheless, STH’s impact becomes more noticeable at lower doses, indicating that it may have chemopreventive qualities in colon cancer. Research on breast and lung cancer cells has shown that MH can inhibit the activity of the cancer-promoting transcription factor p-STAT3. The IL-6 receptor (IL-6R) signaling cascade relies on two key components, gp130 and p-JAK2, which we lower to achieve this. Furthermore, MH and its flavonoid components have been found to bind directly to IL-6Rα. This binding impedes the receptor’s interaction with the IL-6 ligand, highlighting the blockade of IL-6R as a promising therapeutic approach for cancers dependent on IL-6 signaling [15]. Halwani [23] investigates the antimicrobial and anticancer properties of Shaoka and MH on HepG2 and MCF-7 cells. The antibacterial properties of Shaoka honey are comparable to those of phenol on ESBL *Escherichia coli*, and it is especially effective against multi-drug-resistant strains. This honey also has potent apoptotic effects on cancerous cell lines, which raises the possibility that it could be utilized therapeutically to treat cancer and infectious diseases.

The apoptosis pathway is a well-studied mechanism that regulates cell death [24,25]. Cancer cells that avoid apoptosis tend to grow uncontrollably and develop resistance to anti-cancer therapies [26,27]. The majority of chemotherapeutic drugs trigger cell death in cancer by way of the apoptotic pathway, which is regulated by genes belonging to the Bcl-2 family. These genes include both pro- and anti-apoptotic variants, such as Bcl-2 and Bax. The current study showed that LSCF treatment activated apoptosis by increasing Bax expression and decreasing Bcl-2 levels.

Efficient apoptosis requires upregulation of the caspase gene family, and LSCF treatment was found to dose-dependently induce the expression of different caspases, confirming apoptosis activation [28]. These findings are consistent with previous research that showed *L. flavescens* extract induces apoptosis and activates caspase-3 in lung cells [29]. Recent research indicates that MH could possess anti-cancer qualities. It appears to modulate levels of reactive oxygen species within cells and alter intracellular calcium levels, which can lead to the programmed death of cancer cells. This potential anti-cancer mechanism is linked to MH’s distinctive capacity to effectively regulate hydrogen peroxide transfer via aquaporin-3 [30].

The major phytoconstituents found in LSCF were analyzed using GC-MS analysis, which revealed that some of the compounds have anticancer activity. Beta-eudesmol is one of the major components present in LSCF, as shown in Table 2. Many studies have shown that beta-eudesmol triggers caspase 3/7, increases Bax levels, and decreases Bcl-2 levels, leading to cytotoxicity and death in different types of cancer cells [31–33]. This study suggests that these components in CMCF could collaboratively enhance the anti-proliferative properties of *L. scoparium*.

This discovery aligns with Alsaud et al. [5] extraction of β-caryophyllene from manuka leaves using a hydrophobic deep eutectic solvent (HDES) made from menthol and lactic acid. The ethanol extracts had greater quantities of total phenolic components and antioxidant capabilities, according to the research. In contrast, manuka leaf extracts produced by HDES demonstrated significant antibacterial activity, which may have practical applications in industry. Research indicates that deep eutectic solvents (DESs) with a 1:2 molar ratio of menthol to lactic acid extract β-caryophyllene from manuka leaves in New Zealand are better than normal solvents, and environmentally friendly. This extraction method was fine-tuned to the point where it could be reused as a solvent, and under certain conditions, it yielded 10.25 mg of β-caryophyllene per gram of manuka leaf [34].

However, the limitation of this study is that it did not conduct proteomic and metabolomics analysis to investigate the pathway of the anticancer effect of the manuka leaf extract.

**Table 2.** Summary of primary components identified in LSCF using GC-MS.

Compound name	Chemical formula	MW (gm/mol)	RT (min)	Area%
Styrene	C_8_H_8_	104.15	5.87	0.160
2,7-Octadiene-1,6-diol	C_8_H_14_O_2_	142.2	8.06	0.380
Terpinyl acetate	C_12_H_20_O_2_	196.29	10.84	0.200
4-Phenyl-2-butanone (benzyl acetone)	C_10_H_12_O	148.205	11.49	0.420
2-Phenylethyl ester of acetic acid (phenethyl acetate)	C_10_H_12_O_2_	164.204	11.87	0.270
Dihydro-alpha-ionone	C_13_H_22_O	194.31	12.84	0.950
Ylangene	C_15_H_24_	204.35	13.23	1.450
Alpha-copaene (copaene)	C_15_H_24_	204.35	13.35	0.990
Hexyl Ester of benzenepropanoic acid	C_15_H_22_O_2_	234.33	13.58	3.520
Trans-Caryophyllene	C_15_H_24_	204.35	14.10	0.120
Alloaromadendrene	C_15_H_24_	204.35	14.39	1.110
Alpha-muurolene	C_15_H_24_	204.35	14.98	**3.620**
Beta-selinene	C_15_H_24_	204.35	15.20	1.580
Alpha-selinene	C_15_H_24_	204.35	15.29	2.400
1 s-cis-calamenene	C_15_H_22_	202.3352	15.65	**36.440**
Cadina-1,4-diene	C_15_H_22_	204.35	15.79	2.540
Calacorene	‎C_15_H_20_	200.32	15.98	2.190
(-)-Caryophyllene oxide	C_15_H_24_O	220.35	16.59	1.620
2,4,6,8-Tetramethyl-1-nonene	C_13_H_26_	182.35	16.93	3.330
delta cadinol	C_15_H_26_O	222.37	17.16	2.300
Beta-eudesmol	C_15_H_26_O	222.37	17.64	**18.210**
Cyclododecane	C_12_H_24_	‎168.32	18.37	**5.560**
Neophytadiene	C_20_H_38_	278.5	19.30	2.560
2-methyl-6-methylene-octa-1,7-dien-3-ol	C_10_H_16_O	152.23	20.59	3.170
9-octadecenoic acid	C_18_H_34_O_2_	282.5	21.48	0.540

The bold numbers in the table refer to the most significant compounds in the extract.

## Conclusion

Scientific studies have shown that *L. scoparium* chloroform extract can inhibit the development and spread of cancer cells. The extract’s anti-cancer effects are amplified by its association with the induction of apoptosis, which is concentration-dependent. Also, this extract changes a lot of genes that have to do with cell death in these cells. Overall, these results suggest that *L. scoparium* may serve as a valuable resource for developing treatments targeting cancer through apoptotic mechanisms.

## List of Abbreviations

Bax, Bcl-2-associated X protein; Bcl-2, B-cell lymphoma 2; CXP, Cytomics Flow Cytometry (Software by Beckman Coulter); DMEM, dulbecco minimal essential medium; DNA, Deoxyribonucleic Acid; FITC, Fluorescein Isothiocyanate; GCMS, Gas Chromatography-Mass Spectrometry; IC_50_, inhibitory concentration at 50%; LDH, lactate dehydrogenase; LSCF, *L. scoparium* chloroform fraction; mRNA, Messenger Ribonucleic Acid; MCF, Mean Channel Fluorescence; MTT, 3-(4,5-dimethylthiazol-2-yl)-2,5-diphenyltetrazolium bromide; NIST, National Institute of Standards and Technology; PI, Propidium Iodide; RNA, Ribonucleic Acid; SYBR, SYBR Green (a fluorescent dye used in molecular biology).
